# Anti-rheumatoid Activity of Secondary Metabolites Produced by Endophytic *Chaetomium globosum*

**DOI:** 10.3389/fmicb.2016.01477

**Published:** 2016-09-20

**Authors:** Ahmed M. Abdel-Azeem, Sherif M. Zaki, Waleed F. Khalil, Noha A. Makhlouf, Lamiaa M. Farghaly

**Affiliations:** ^1^Botany Department, Faculty of Science, Suez Canal UniversityIsmailia, Egypt; ^2^Microbiology Department, Faculty of Science, Ain Shams UniversityCairo, Egypt; ^3^Pharmacology Department, Faculty of Veterinary Medicine, Suez Canal UniversityIsmailia, Egypt; ^4^Histology Department, Faculty of Medicine, Ain Shams UniversityCairo, Egypt; ^5^Histology Department, Faculty of Medicine, Suez Canal UniversityIsmailia, Egypt

**Keywords:** *Chaetomium globosum*, adjuvant-induced arthritis, arid Sinai, fungarium, saint katherine protectorate

## Abstract

The aim of the present study was to investigate the anti-rheumatoid activity of secondary metabolites produced by endophytic mycobiota in Egypt. A total of 27 endophytic fungi were isolated from 10 dominant medicinal plant host species in Wadi Tala, Saint Katherine Protectorate, arid Sinai, Egypt. Of those taxa, seven isolates of *Chaetomium globosum* (CG1–CG7), being the most frequent taxon, were recovered from seven different host plants and screened for production of active anti-inflammatory metabolites. Isolates were cultivated on half – strength potato dextrose broth for 21 days at 28°C on a rotatory shaker at 180 rpm, and extracted in ethyl acetate and methanol, respectively. The probable inhibitory effects of both extracts against an adjuvant induced arthritis (AIA) rat model were examined and compared with the effects of methotrexate (MTX) as a standard disease-modifying anti-rheumatoid drug. Disease activity and mobility scoring of AIA, histopathology and transmission electron microscopy (TEM) were used to evaluate probable inhibitory roles. A significant reduction (*P* < 0.05) in the severity of arthritis was observed in both the methanolic extract of CG6 (MCG6) and MTX treatment groups 6 days after treatment commenced. The average arthritis score of the MCG6 treatment group was (10.7 ± 0.82) compared to (13.8 ± 0.98) in the positive control group. The mobility score of the MCG6 treatment group (1.50 ± 0.55) was significantly lower than that of the positive control group (3.33 ± 0.82). In contrast, the ethyl acetate extract of CG6 (EACG6) treatment group showed no improvements in arthritis and mobility scores in AIA model rats. Histopathology and TEM findings confirmed the observation. Isolate CG6 was subjected to sequencing for confirmation of phenotypic identification. The internal transcribed spacer (ITS) 1–5.8 s – ITS2 rDNA sequences obtained were compared with those deposited in the GenBank Database and registered with accession number KC811080 in the NCBI Database. The present study revealed that the methanol extract of endophytic fungus *C. globosum* (KC811080) recovered from maidenhair fern has an inhibitory effect on inflammation, histopathology and morphological features of rheumatoid arthritis in an AIA rat model.

## Introduction

Endophytic fungi are symbiotically associated biota of living plant tissues that induce symptomless disease to their hosts (Petrini, 1991) and are non-host specific (Cohen, 2006). Over last decade, scientists have focused their investigations on bio prospecting naturally occurring chemical compounds and biological material, especially in extreme diverse environments (Suryanarayanan et al., 2009; Abdel-Azeem et al., 2012; Mustafa et al., 2013; Salem and Abdel-Azeem, 2015). Medicinal plants and microbiota are the most consistent and generative sources of ‘first-in-class’ drugs (Newman and Cragg, 2007). Recently, remarkable pharmacological agents have been generated from endophytic fungi (Strobel and Daisy, 2003). More than 50% of previously unknown biologically active substances have been isolated from endophytes (Schulz et al., 2002). Endophytes have been the source of a number of bio-pharmacological compounds including those with antimicrobial, antitumor, anti-inflammatory, and antiviral activities (Aly et al., 2008; Liu et al., 2008; Souza et al., 2008). In Egypt, endophytic fungi from aquatic, halophilic, medicinal plants, and marine resources have been studied by various investigators (El-Morsy, 2000; Abdel-Motaal et al., 2010; Aly et al., 2011; Selim et al., 2011; Abdel-Monem et al., 2013; Salem and Abdel-Azeem, 2014).

*Chaetomium* Kunze is a cosmopolitan genus with about 100 accepted species (Kirk et al., 2008). In Egypt, 53 species and one variety of the genus *Chaetomium* have been recorded (Moustafa and Abdel-Azeem, 2005). *Chaetomium* has attracted the attention of researchers as an important genus in Ascomycota because of the variety of biological and biotechnological applications of its species in different areas, e.g., medical mycology (Zhang et al., 2010), biotechnology (Soni and Soni, 2010), and molecular studies (Aggarwal et al., 2008). To the best of our knowledge, more than 200 compounds, associated with unique and diverse structural types have been isolated and chemically identified from the genus *Chaetomium* (Fujimoto et al., 2004; Jiao et al., 2004; Bashyal et al., 2005; Kobayashi et al., 2005; Ding et al., 2006; Isham et al., 2007; Selim et al., 2014).

Rheumatoid arthritis is an autoimmune disease of humans that characterized by chronic inflammation of the synovial joints and erosive destruction of articular tissue due to progressive inflammation (Ngian, 2010). About 0.5–1% of the human population worldwide is affected by RA and 20–50 cases per 100,000 are recorded annually (Karmakar et al., 2010). MTX had become the principal drug used for the treatment of RA (Williams et al., 1985). MTX is an antifolate immunosuppressive drug that acts primarily on highly proliferating cells, during the synthesis (S)-phase of the cell cycle and inhibits neutrophil chemotaxis (Moreland et al., 1997). Treatment with MTX has been limited because of its toxicity and adverse side effects such as cytopenia, bloody vomit, diarrhea, nephrotoxicity and alopecia (Alarcon et al., 1989). Hence, the discovery of new drugs for the treatment of RA has become a major target of potentially considerable value.

New anti-inflammatory agents produced by fungi have been the focus of a few studies conducted by several investigators over the last two decades (Matsumoto et al., 1995; Chapuis et al., 2000; Lull et al., 2005; Schmidt et al., 2012). In order to fill-gaps in the research area of anti-inflammatory properties of fungal metabolites, we investigated the capability of endophytic mycobiota from wild medicinal host plants in the Saint Katherine Protectorate, Egypt, to produce anti-rheumatoid arthritis metabolites, and their probable inhibitory effects in an AIA rat model compared to the effects of MTX a standard disease-modifying anti-rheumatoid drug.

## Materials and Methods

### Study Area and Sampling

Wadi Tala (1450–1670 m above sea level) is a rocky U-shaped valley, running from North to South, approximately 2.5 km west of Saint Katherine city. One hundred samples of the dominant plant species from ten localities namely: *Artemisia herba-alba* Asso; *Achillea fragrantissima* (Forssk) Sch.; *Capparis spinosa* L.; *Chiliadenus montanus* (Vahl) Brullo; *Echinops spinosissimus* Turra; *Origanum syriacum* L.; *Phlomis aurea* Decne; *Teucrium polium* L.; *Verbascum sinaiticum* Benth.; and *Adiantum capillus-veneris* L. were collected in sterilized polyethylene bags and transferred to the laboratory, where they were subsequently plated out. Samples were collected under permission of the Saint Katherine Protectorate for scientific purposes and no endangered species were involved in the study.

### Isolation of Endophytic Mycobiota

A total of 1000 plates were used for the isolation of endophytic mycobiota (100 plates/plant). Pieces of stem and leaves (5 mm^2^, four pieces in each plate) were surface sterilized and cut. The sections were washed three times in running water, immersed in 70% ethanol for 1–5 min, dipped in 5% NaOCl for 3–5 min, according to the plant thickness, and then dipped in 70% ethanol for 0.5 min (Fisher et al., 1993), before being plated on appropriate isolation media. For primary isolation, Czapek’s yeast extract agar, supplemented with Rose bengal (1/1500), chloramphenicol (50 ppm), and Potato Dextrose Agar media were used.

### Phenotypic Identification

Identification of the recovered endophytic fungal isolates was conducted up to the species level based on phenotypic means was and the relevant identification keys for: *Penicillium* (Raper and Thom, 1949; Pitt, 1980) *Aspergillus* (Klich, 2002); dematiaceous hyphomycetes (Ellis, 1971, 1976); *Fusarium* (Booth, 1971); miscellaneous fungi (Domsch et al., 2007); ascomycetes (Guarro et al., 2012) and *Chaetomium* (Doveri, 2013). The names of the authors of fungal taxa were abbreviated according to Kirk and Ansell (1992). The systematic arrangement of the recorded list follows the 10th edition of Ainsworth and Bisby’s Dictionary of the Fungi (Kirk et al., 2008). All name corrections, authorities, and taxonomic assignments of recorded species in the present study were checked against the Index Fungorum database.

### Molecular Identification

Molecular identification of the promising isolate, *Chaetomium globosum* (CG6), was performed by comparing its ITS1 – 5.8S – ITS2 rDNA region sequence data with data on reference strains deposited in GenBank.

### DNA Isolation

The Fungal isolate was grown on Potato dextrose agar and DNA was extracted according to the protocol provided in the Fermentas^®^ Genomic DNA Purification Kit #K0512 (Thermo Fischer Scientific, Europe). Sufficient inoculum was suspended in 200 μL Tris-EDTA buffer (10 mM Tris-HCl, pH 8.0, 1 mM EDTA) in a 2.2 ml Eppendorf tube. The tubes of each sample were boiled for 3 min and then placed in ice water for 10 min. Lysis solution (400 μL) was added and the tubes were incubated at 65°C for 30 min, after which 600 μL of chloroform was added and the resulting solution carefully mixed. DNA was separated by centrifugation at 12000 rpm for 10 min at 4°C, and mixed with 800 μL of precipitation solution through several inversions at room temperature for 1 min. The tubes were then centrifuged at 12000 rpm for 10 min at 4°C. Pellets of DNA were dissolved in 100 μL of 1.2 M NaCl solution by gentle vortexing. Ice-cold isopropanol (500 μL) was added to the solution and each tube was incubated for 15 min at -20°C and then centrifuged at 12000 rpm for 10 min at 4°C. The DNA pellet was washed with 1 mL of ice-cold 70% ethanol, dried, and resuspended in sterile Tris-EDTA buffer.

### Oligonucleotides

The oligonucleotide primers described by White et al. (1990) were used for amplification and sequencing of the ITS regions. ITS5 (5′-GGAAGTAAAAGTCGTAACAAGG-3′) AND ITS4 (5′-TCCTCCGCTTATTGATATGC-3′) (Bioneer Corporation, South Korea) were selected for the present study.

### PCR and DNA Sequencing of the ITS1 – 5.8 s – ITS2 rDNA Region

Amplification reactions were carried out in 20 μL reaction mixtures containing 2.5 μL of each primer (10 pm), 2.5 μL of genomic DNA (5 μg/mL), and one PCR-Gold Master-Mix bead (Bio-ron, Germany). The bead contained buffers, dNTP, an enzyme, stabilizers, Tris-HCl, KCl, and MgCl2. A PCR Thermal Cycler (Techne^®^Genius – England) was used for amplification at the following settings: initial denaturation at 96°C for 5 min, 35 cycles of denaturation at 94°C for 30 s, annealing at 52°C for 30 s, extension at 72°C for 80 s, and a final extension at 72°C for 10 min. Products of the PCR reaction were sequenced directly using the Big-Dye terminator reagent kit and Taq polymerase in an automated DNA sequencer (Model 3100; PerkinEl-mer Inc/Applied Biosystems – Bioneer, South Korea), according to the manufacturer’s protocol.

### Nucleotide Accession Number

The nucleotide sequence data of the CG6 isolate of the present study was deposited in the NCBI GenBank nucleotide sequence database under accession number KC811080.

### Extraction of Active Metabolites from Recovered *C. globosum* Isolates

Isolates of *C. globosum* under investigation (CG1–CG7) were grown on Oat Meal Agar at 28°C for 15 days. Each isolate was prepared by inoculation in 2 L Erlenmeyer flasks containing 1 L autoclaved potato dextrose broth and shaking at 180 rpm at 28°C for 21 days. The fermentation broth of each isolate was divided into two portions (2 L each) and filtered. Fresh mycelia were washed three times with distilled water and stored in a freezer. Two organic solvents, namely ethyl acetate and methanol, were used for extraction of active metabolites. The filtrate was divided into two portions (2 L each), and extracted three times with equal volumes of ethyl acetate and methanol, and collected separately. The frozen mycelia were ground and extracted three times in each solvent, and combined with organic extracts of the filtrate and evaporated until dry under reduced pressure according to the procedures outlined by Salem and Abdel-Azeem (2014). After evaporation, the dried extract was stored in away from light in a refrigerator until further use. For injection of rats, fresh prepared solution of solid metabolites was applied through re-suspension in sterile 10% Tween-80 in saline solution.

### Animals, Induction of Adjuvant Induced Arthritis (AIA) Rat Model, and Treatments

Male Wistar albino rats (102) weighing 160–180 g were obtained from the Animal House Colony of the National Research Center of Egypt and divided into five groups (six rats each) after a week of acclimatization. The first group was the negative control group (NC) that was injected with saline and 10% Tween-80, instead of CFA and fungal extracts, respectively. All other groups were injected subcutaneously at the base of the tail with 100 μL CFA (Sigma-Aldrich, USA) to induce arthritis (Bendele, 2001). The second group was the positive control group (PC) that remained untreated but was administered 10% Tween-80 vehicle alone. The third and fourth groups (seven replicates each) were injected with methanol (MCG) and ethyl acetate (EACG) fungal extracts, respectively. The fifth group was treated with MTX (Orion Pharma, Espoo, Finland) as a standard disease-modifying anti-rheumatic-drug. All possible efforts were made to minimize animal suffering and reduce the number of rats used. The experimental protocol was approved by the Scientific Research Ethics Committee of the Faculty of Veterinary Medicine, Suez Canal University. After 14 days of CFA administration, clinical signs of arthritis were clearly evident and all treatments commenced at that time. Ethyl acetate and methanol fungal extracts were injected subcutaneously twice per week for 2 weeks at a dose of 10 and 30 μg extract/Kg BW., respectively, based on the finding of a pilot study conducting in the veterinary pharmacology laboratory of Suez Canal University. Similarly, MTX was injected subcutaneously twice per week at a dose of 0.3 mg/Kg BW (Suzuki et al., 1997). The lowest doses that exhibited curative effects without apparent toxicity were selected for further analysis.

### Assessing Swelling and Mobility Scoring

On the first day of treatment, swelling was assessed in the right hind paw via measurement of its mean thickness using a 0–10 mm electric caliper. Four definitions were used to score animal mobility according to the scale proposed by Ablin et al. (2010). The scores ranged from 0 to 4 as follows: 0 = normal, 1 = slightly impaired, 2 = major impairment, 3 = does not bear weight on paw, and 4 = no movement. Measurements and scoring of arthritis’s were performed independently by two blinded technicians.

### Histopathology Studies

Rats were sacrificed under light ether anesthesia and hind limbs were resected and fixed in 10% buffered formalin. Limbs were decalcified in 5% nitric acid, dehydrated, cleared, and embedded in paraffin for sectioning at a thickness of 5 μm. Sections were subsequently stained with hematoxylin and eosin (H & E; Bancroft and Stevens, 1996).

### Transmission Electron Microscope Examination

Samples of skin, muscle, fatty tissues, and tendons from sacrificed rats were removed, trimmed of excessive subchondral bone, and cut into 1 mm^3^ slabs. Fixation of cartilages, decalcification, rinsing, post-fixation, dehydration, embedding, sectioning, and ultra-sectioning were carried out. Ultra-thin sections were stained according to the methods outlined by Bancroft and Gamble (2008), using uranyl acetate and lead citrate. They were subsequently examined under a TEM (JEOL 1200 EX II, Japan) at the regional Mycology and Biotechnology center, AL-Azhar University, Egypt.

### Data Analysis

Data of the treated groups were compared with those of the PC group (untreated AIA) to determine the significance of treatment efficacy. Data were subjected to the Bartlett’s test (Bartlett, 1937; Winer et al., 1991), ANOVA, and Dunnett’s multiple comparisons (Hsu, 1996). If unequal variances were obtained in the Bartlett’s test, data were subjected to the non-parametric Kruskal–Wallis test for comparisons between treatments and the PC group (Daniel, 1990). Significance between groups was accepted when *P* < 0.05.

## Results

### Species Composition

A total of 27 species, belonging to 19 genera of endophytic mycobiota, associated with 10 dominant plant species along Wadi Tala were recorded (**Table 1**). The results showed that teleomorphic Ascomycota were represented by four species (14.82%) and anamorphic Ascomycota by 23 species (85.18 %). *Aspergillus* (four species; 14.82%), *Chaetomium* (three species; 11.11%), *Alternaria* (three species; 11.11%), *Penicillium* (two species; 7.41%), and the remaining genera each represented by only one species were detected. Among all endophytic species recorded, *C. globosum* represented the most prevalent endophyte isolated (22.34% of the total number of isolates per plate) followed by *Alternaria alternata* (19.32%), *Nigrospora oryzae* (16.77%), and *Sarocladium strictum* (8.72%).

**Table 1:** Total count, number of cases of isolation, and frequency of fungal taxa recovered.

Species	TC^∗^	NCI^∗∗^	% F^∗∗∗^
**Ascomycota (teleomorphic)**			
*Chaetomium bostrychodes* Zopf	13	6	0.6
*Ch. Globosum* Kunze	333	112	11.2
*Ch. piluliferum* J. Daniels	17	5	0.5
*Thielavia terricola* (J.C. Gilman & E.V. Abbott) C.W. Emmons	30	4	0.4
**Ascomycota (anamorphic)**			
*Acremonium rutilum* W. Gams	130	55	5.5
*Alternaria alternate* (Fr.) Keissl.	288	250	25
*A. atrum* (Preuss) Woudenberg & Crous	12	4	0.4
*A. tenuissima* (Kunze) Wiltshire	27	6	0.6
*Aspergillus candidus* Link	6	3	0.3
*A. flavus* Link	35	14	1.4
*A. niger* Tiegh.	42	28	2.8
*A. terreus* Thom	17	5	0.5
*Botryotrichum piluliferum* Sacc. & Marchal	7	7	0.7
*Cladosporium cladosporioides* (Fresen.) de Vries	13	9	0.9
*Curvularia lunata* (Wakker) Boedijn	7	3	0.3
*Drechslera spicifera* (Bainier) Arx	90	45	4.5
*Fusarium solani* (Mart.) Sacc.	45	15	1.5
*Nigrospora oryzae* (Berk. & Broome) Petch	250	230	23
*Penicillium chrysogenum* Thom	19	5	0.5
*Penicillium rubrum* Stoll	3	2	0.2
*Phoma herbarum* Westend.	14	7	0.7
*Sarocladium strictum* (W. Gams) Summerb.	55	25	2.5
*Stachybotrys chartarum* (Ehrenb.) S. Hughes	5	3	0.3
*Stemphylium botryosum* Sacc.	7	3	0.3
*Trichothecium roseum* (Pers.) Link	5	3	0.3
*Trichoderma pseudokoningii* Rifai	12	3	0.3
*Ulocladium botrytis* Preuss	8	3	0.3
Total	1490		

*^∗^Colonies/Cut; ^∗∗^Number of Cases of Isolation; ^∗∗∗^Frequency.*

### Phenotypic Identification of *C. globosum* Isolates

Seven isolates of *C. globosum* were morphologically identified. Colonies showed a daily growth rate of 7–8 mm, with pale or olivaceous aerial mycelia, often with yellow, gray–green, green or red exudates. Ascomata mature within 7–9 days, measured 175–280 μm, and were olivaceous, gray–green or brown in reflected light, and tended to be superficial, spherical, ovate or obovate, and ostiolate. The ascomatal wall was brown in color and composed of textura intricate. The cells were 2.0–3.5 μm in breadth, and the ascomatal hairs were numerous, typically unbranched, flexuous, undulate or coiled, often tapering, septate, brownish, 3–4.5 μm in breadth at the base, and up to 500 μm in length. The asci were clavate or slightly fusiform, stalked, evanescent, measured 30–40 × 11–16 μm, and contained eight ascospores. Ascospores were limoniform, typically biapiculate, bilaterally flattened, brownish when mature, thick-walled, contained numerous droplets, measured 9–12 × 8–10 × 6–8 μm, and featured an apical germ pore. Paraphyses were not observed.

### Therapeutic Effects of Secondary Metabolites of *C. globosum* on Disease Activity and Mobility Scores

**FIGURE 1:**
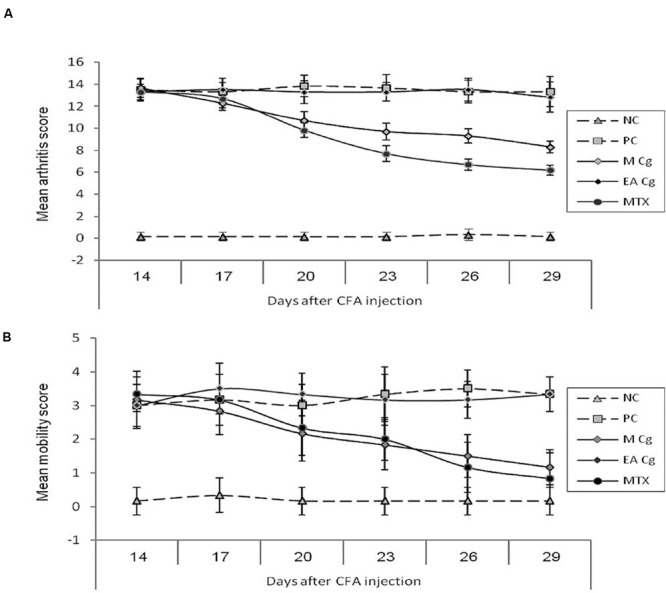
**Effect of *Chaetomium globosum* methanol (MCg) and ethyl acetate (EACg) extracts on the arithritis (graph **A**-mean arithritis score) and mobility scores (graph **B**-mean mobility score) after 14, 17, 20, 23, 26, and 29 days from arithritis induction.** Mobility scale represented as 0 = normal, 1 = slightly impaired, 2 = major impairment, 3 = does not step on paw, and 4 = no movement.

Severe arthritis was clearly evident in rats by day 14 after subcutaneous injection of CFA at the base of the tail, and persisted for more than 32 days. Treatment of AIA rats with the methanolic extract (MCG6) resulted in a significant reduction (*P* < 0.05) in severity of the arthritis score in comparison to the untreated PC. This curative effect was observed in both the MCG6 and MTX groups, 6 days after treatment commenced. The anti-arthritic effect of MCG6 increased gradually until the end of the experiment, 29 days post CFA administration (**Figure 1A**).

At 20 days post CFA administration, the average arthritis score of MCG6 treated rats was 10.7 ± 0.82 (mean ± standard deviation) compared to 13.8 ± 0.98 in PC rats. In contrast, the rats treated with ethyl acetate extract of CG6 (EACG6) showed no improvement in arthritis score through out the experimental period (no significant differences were observed between EACG6 and PC groups).

A significant reduction in the mobility score was observed following treatment with MCG6 indicating a clinical improvement in joint function. This reduction was significantly lower than that of PC rats on day 26 (1.50 ± 0.55 and 3.33 ± 0.82 for MCG6 and PC groups, respectively), and this significance persisted (*P* < 0.05) until the end of the treatment period at 29 days post CFA administration. The EACG6 group again showed no improvement in mobility scores and no significant differences were observed between the EACG6 and PC groups (**Figure 1B**).

### Histopathology Findings

**FIGURE 2:**
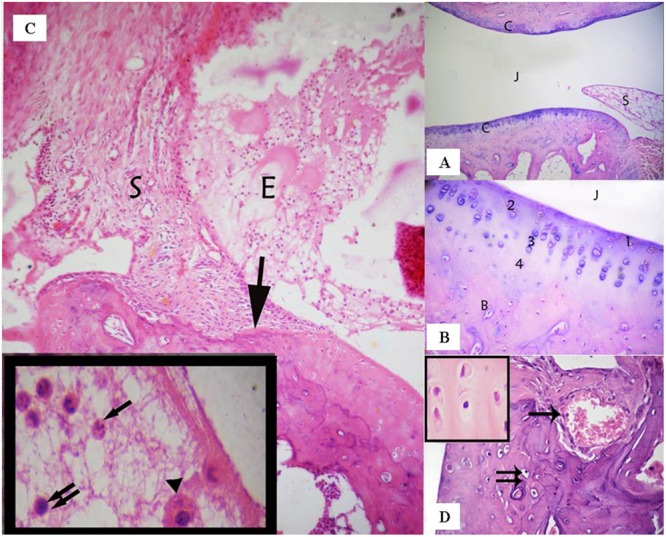
**(A)** Ankle joint of the control group showing the articular cartilage **(C)**, the joint cavity (J), and the synovial membrane (S). (Control group, H & E, ×100). **(B)** Articular cartilage with its four zones: a superficial tangential zone (1), a transitional zone (2) that contained scattered chondrocytes, a radial zone (3) containing chondrocytes arranged perpendicular to the surface. A calcified zone (4) separating hyaline cartilage from the underlying subchondral bone **(B)**. (Control group, H & E, ×400). **(C)** A photomicrograph of the rheumatoid arthritis group showing infiltration of the synovial tissue (S) by inflammatory cells- that grows over the articular cartilage (↑) and causes its erosion and irregularity. The nearby articular cartilage has lost its basophilia. The joint cavity is filled with inflammatory cells and exudation (E). Inset: fluid filling the joint cavity contains fibrin deposits and many inflammatory cells, including polymorphonuclear leukocytes (↑), lymphocytes (↑↑), and macrophages (▲). (Arthritis group H & E, ×100, Inset ×1000). **(D)** A photomicrograph of arthritis group showing invasion of the cartilage matrix by fibrovascular synovial tissue that is compressing the underlying bone. Note: Blood vessels have invaded the cartilage matrix (↑). Notice also apoptotic chondrocytes (↑↑). Inset shows the acidophilic matrix of cartilaginous tissue and chondrocytes with darkened nuclei and shrunken cytoplasm; some chondrocytes have eccentric nuclei. (Arthritic group, H & E, ×400; Inset×1000).

Sections of the control group that had been stained with (H & E) showed that the ankle joint was covered with typical hyaline cartilage (articular cartilage) on both surfaces, lacked a perichondrium and was separated by a joint cavity filled with articular fluid (**Figure 2**). Four zones were identified in the articular cartilage as follows: superficial tangential (with elongated chondrocytes and a long axis that was parallel to the surface), transitional (middle zone that contained scattered rounded chondrocytes), radial (with spherical chondrocytes arranged perpendicular to the surface), and calcified (that separated hyaline cartilage from the underlying subchondral bone) (**Figure 2A**).

The synovial membrane thickened and became infiltrated by inflammatory cells in the arthritic group. The thickened areas extended over, and penetrated deep into the articular cartilage, to form what is referred to as pannus and thereby, causing erosion and irregularity of the cartilage. The joint cavity was filled with exudation and inflammatory cells that included polymorphonuclear leukocytes, lymphocytes, and macrophages. The matrix of the nearby articular cartilage exhibited loss of basophilia (**Figure 2B**). Vascular synovium penetrated the cartilage and compressed the underlying bone. The chondrocytes were shrunken with darkened nuclei that were sometimes eccentric (**Figure 2C**).

The synovium was infiltrated with macrophages, lymphocytes, plasma cells, and fibroblast - like spindle cells that represented a mononuclear cellular inflammatory infiltrate. Plasma cells had eccentric nuclei and pink cytoplasm containing Russell bodies. As a prominent feature of the synovium was the presence of hyperplasia of spindle-shaped cells (**Figure 2D**).

**FIGURE 3:**
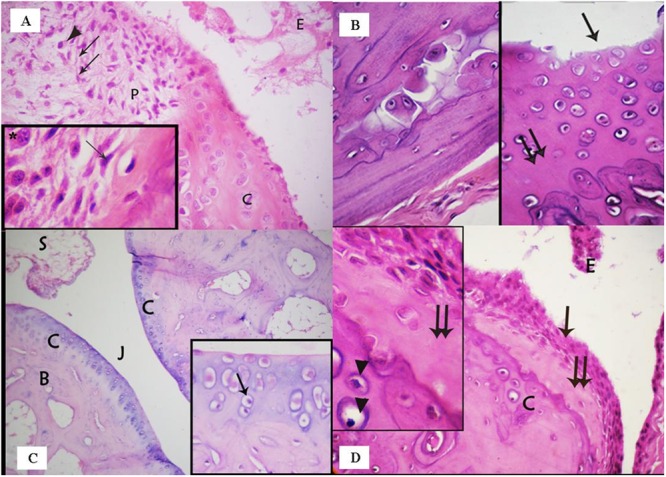
**(A)** photomicrograph of the rheumatoid arthritis group showing infiltration of the synovial tissue by fibroblast – like spindle shaped cells (↑) and inflammatory cells, e.g., plasma cells (▲). Note that the nearby articular cartilage (C) has lost its basophilia. Notice also inflammatory cells and exudate (E) in the joint cavity. Inset: higher magnification of spindle shaped cells (↑) and plasma cells (^∗^) in the synovium. (Arthritis group, H & E, ×400; Inset ×1000). **(B)** Photomicrograph of arthritis group showing: (a) areas of bone destruction within the bone matrix; (b) irregular surface of the articular cartilage with erosion on the surface (↑) and loss of its smooth contour. Note also areas of cell loss (↑↑). (Arthritis, H & E, ×400). **(C)** Photomicrograph of the ankle joint of the group treated with a methanolic extract of the fungus, showing the articular cartilage (C), the joint cavity (J), and synovial membrane (S). Few degenerated cells are evident (↑). (Methanolic extract group. H & E, ×100; Inset ×400). **(D)** Photomicrograph of the group treated with an ethanolic extract of the fungus showing the articular cartilage (C) covered by hyperplastic synovium (↑). The underlying cartilage has lost its basophilia and many chondrocytes appear degenerated. Inset: acidophilic matrix, area of cell loss (↑↑), and apoptotic chondrocytes (▲). (Ethyl acetate extract group, H & E, ×400; Inset×1000).

The articular cartilage and bone beneath and beside the pannus were disrupted and areas of bone destruction were detected in the juxta-articular region (**Figure 3A**). The surface of the articular cartilage showed irregular textur, with surface erosions, and a loss of smooth contours (**Figure 3B**). The surrounding cartilage was characterized by a loss of basophilia in the matrix and degenerated chondrocytes. Many chondrocytes appeared shrunken with acidophilic cytoplasm and pyknotic nuclei, vacuolated cytoplasm or darkened eccentric nuclei (**Figures 3C,D**). Various areas in the articular cartilage showed cell loss (**Figures 2D** and **3B**). The changes observed in the articular cartilage occurred peripherally and extended toward the center.

Examination of the MCG6 group showed that the ankle joint was covered by articular cartilage that was observed to be similar to that of the negative control group, except for the presence of some degenerated chondrocytes. The synovium was devoid of inflammatory cell infiltrates and the joint cavity was free of any exudates or inflammatory cells (**Figure 3C**).

Microscopic inspection of the EACG6 group showed that the synovium was hyperplastic and grew over the articular cartilage. The nearby cartilage exhibited an acidophilic matrix, some degenerated chondrocytes and areas of cell loss. The joint cavity showed an accumulation of cells and exudates without fibrin deposits (**Figure 3D**).

### TEM Examination

**FIGURE 4:**
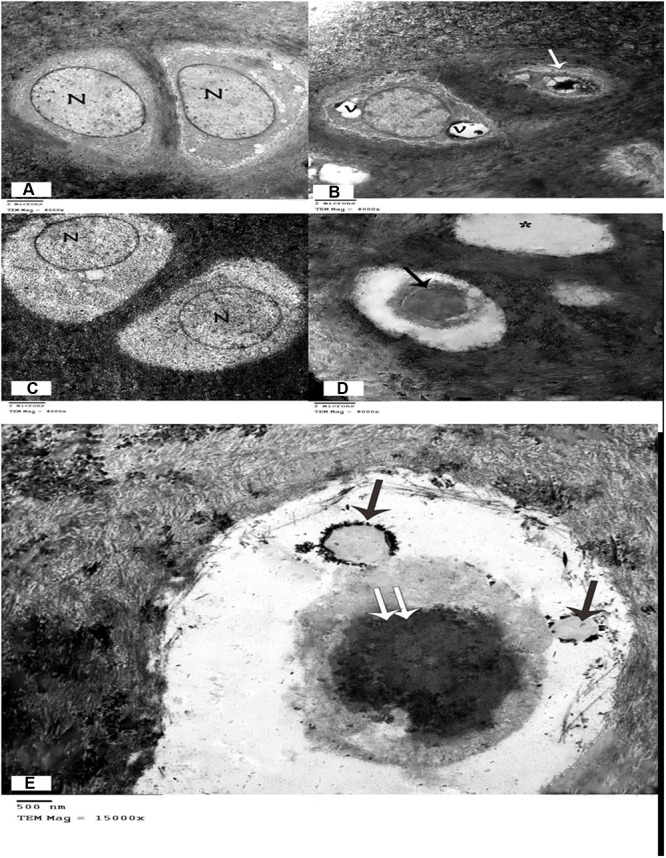
**(A)** Electron micrograph showing two chondrocyte with lacunae. Vesicular nuclei (N), and cytoplasm with few organelles are evident. Chondrocytes are surrounded by a matrix full of collagen fibrils. (Control group, TEM, ×8000). **(B)** Electron micrograph of the arthritic group showing one chondrocyte with large vacuoles in the cytoplasm and another chondrocyte that appears shrunken with irregular contours, scanty cytoplasm, and a dark, irregular nucleus. (Arthritis group, TEM, ×8000). **(C)** Electron micrograph showing two chondrocytes within lacunae with vesicular nuclei and normal cytoplasm. (Group treated with methanolic extract of the fungus, TEM, ×8000). **(D)** Electron micrograph of the group treated with an ethyl acetate extract of the fungus, showing one shrunken chondrocyte with dark nucleus (↑), and empty lacuna (^∗^). (Methanolic extract of the fungus group, TEM, ×8000). **(E)** Electron micrograph of the group treated with a methanolic extract of the fungus, showing apoptotic bodies (↑) in a shrunken chondrocyte with dark nucleus (↑↑). (Methanolic extract of the fungus group, TEM, ×15000).

Examination of TEM micrographs of the control group revealed that the chondrocytes had large vesicular nuclei, surrounded by faint cytoplasm with few organelles. The capsular or territorial zone that defines the matrix surrounding the cells, contained an abundance of randomly arranged collagen fibrils (**Figure 4A**). Degenerative changes were detected in the chondrocytes, including irregular contours, atrophied cell bodies, scanty cytoplasm, loss of cell processes, and dark irregular nuclei or vacuolated cytoplasm with many empty lacunae (**Figure 4B**). Examination of the MCG6 group revealed well-preserved chondrocytes with vesicular nuclei similar to those of the control group (**Figure 4C**). TEM studies of the EACG6 group showed shrunken chondrocytes with dark nuclei and apoptotic bodies. Many empty lacunae were also detected (**Figures 4D,E**).

### Molecular Identification of the *C. globosum* (CG6) Isolate

The sequences of the ITS1–5.8 s–ITS2 rDNA region of the *C. globosum* (CG6) isolate were 510 bp in length. The NCBI database was accessed to identify the isolate using the BLAST homology search and the obtained ITS data. The ITS data of the isolated *C. globosum* (CG6) isolate was 99% identical to GenBank data of *C. globosum* (GenBank Accession Number JN209920).

## Discussion

Endophytic fungi represent an important factor in improving the drug discovery process, as they might consistently exhibit antimicrobial, anticancer, antiviral, and antioxidant activities (Strobel, 2002; Firáková et al., 2007; Debbab et al., 2011; Salem et al., 2013; Salem and Abdel-Azeem, 2014). Recently, anti-inflammatory and anti-thrombotic effects of a metabolite produced by Ascomycete fungal species were reported by Bollmann et al. (2015). Data of the present study regarding endophytic fungi showed that counts of fungal populations were relatively moderate. Similar observations of moderate fungal counts associated with medicinal plants from the Saint Katherine Protectorate have been recorded by several investigators (Selim et al., 2011, 2014; Abdel-Azeem and Salem, 2012). In comparison to endophytic taxa that have been previously isolated from the Saint Katherine Protectorate, our data indicates that some fungi are common to some species of medicinal plants, e.g., *C. globosum, Alternaria alternata*, and *Nigrospora oryzae*. These associations could be attributed to the chemical constituents of the plants. The ability of some of these plant species to live under water stress and the presence of various chemical compounds have been proven on endophytic actinomycetes by El-Shatoury et al. (2013) and on endophytic fungi in Saint Katherine Protectorate by Salem and Abdel-Azeem (2014). Tan and Zou (2001) reported that it is possible to isolate hundreds of endophytic species from a single plant, with at least one of those species generally showing host specificity.

Rheumatoid arthritis is a severe, widespread disease that affects the joints of all age groups. Results of the present study showed a significant reduction (*P* < 0.05) in mobility scores and arthritic changes in both the MCG6- and MTX- treated AIA groups, in comparison to the PC group, whereas the EACG6 extract failed to either reduce or increase these scores. MTX was used as a first-line standard drug for the treatment of RA. The MCG6 dose administered in the present study (10 μg/Kg BW., twice weekly for 2 weeks) has for the first time been proven to significantly ameliorate histological features of the disease, joints inflammation, and severity of arthritis and improve motility as confirmed by histological and electron microscopic assessments. Joint exudates, inflammatory infiltration, pannus formation, synovial hyperplasia, cartilage degradation, and destruction of bone were all considerably reduced. Similarly, Gunatilaka (2006) stated that bioactive metabolites extracted from endophytes could be used as novel sources of antibiotics, immunosuppressants, antiparasitics, antioxidants, and anticancer agents. As RA is considered a reactive oxygen species (ROS)-linked disease (Valko et al., 2007), the beneficial effects of MCG6 might be due to its anti-oxidant properties that effectively combat the damage caused by ROS and oxygen – derived free radicals. Various types of biochemical compounds have been produced by *C. globosum*, including chaetoglobosins (cha; Zhang et al., 2010). Chaetoglobosins have anti-inflammatory properties and have been observed to significantly inhibit the production of tumor necrosis factor TNF-α, interleukin 6 (IL-6) and monocytes chemotactic protein-1 (MCP-1) (Dou et al., 2011). Hua et al. (2013) indicated that the cha-F metabolite has immunosuppressive properties that might prove useful in the control of dendritic cells associated with autoimmune and/or inflammatory diseases.

In the present study, the methanolic extract was found to be more effective than the ethyl acetate extract. Our results are consistent with those of Liu et al. (2007), who evaluated the antioxidant activity of the methanolic extract of endophytic *Xylaria* sp. isolated from *Ginkgo biloba*. The results indicated that in comparison to the ethyl acetate extract, the methanolic extract exhibited strong antioxidant activity, owing to the presence of phenolics and flavonoids. One host plant of *C. globosum* (CG6), *A. capillus-veneris*, contains many anti-inflammatory substances (Haider et al., 2013). The ability of this isolate (CG6) to produce anti-inflammatory substances could be attributed to its long period of co-evolution with *A. capillus-veneris*. This ability can also be expressed as the ability to produce the same or similar bioactive compounds as those produced by the host plants (Zhao et al., 2010, 2011).

## Conclusion

In an AIA rat model that considered morphological, inflammatory, and histopathological features, metabolites of an endophytic native isolate of *C. globosum* (KC811080), recovered from maidenhair fern exhibit a direct inhibitory effects on RA. The present study highlights the remarkable use of fungal technology to produce potentially valuable products (anti-rheumatoid drugs), provides strong scientific evidence to the folkloric uses of this plant in the treatment of RA, and is interesting from a conservationist point of view, as isolated native endophytic taxa are maintained in the Fungarium of ASFC. We recommend further chemical studies to isolate the active principles of the extract of *C. globosum* evaluated in the present study.

## Author Contributions

All authors listed, have made substantial, direct, and intellectual contribution to the work, and approved it for publication.

## Conflict of Interest Statement

The authors declare that the research was conducted in the absence of any commercial or financial relationships that could be construed as a potential conflict of interest.

## Abbreviations

AIA: adjuvant induced arthritis
ASFC: Arab society of fungal conservation
CFA: complete Freund’s adjuvant
ITS: internal transcribed spacer
MTX: methotrexate
NC: negative control
NCBI: National Center for Biotechnology
PC: positive control
RA: rheumatoid arthritis
TEM: transmission electron microscope
